# The contribution of water radiolysis to marine sedimentary life

**DOI:** 10.1038/s41467-021-21218-z

**Published:** 2021-02-26

**Authors:** Justine F. Sauvage, Ashton Flinders, Arthur J. Spivack, Robert Pockalny, Ann G. Dunlea, Chloe H. Anderson, David C. Smith, Richard W. Murray, Steven D’Hondt

**Affiliations:** 1 Graduate School of Oceanography, University of Rhode Island, Narragansett, RI USA; 2 United States Geological Survey, Hawaiian Volcano Observatory, Hilo, HI USA; 3 Woods Hole Oceanographic Institution, Woods Hole, MA USA; 4 MARUM - Center for Marine Environmental Sciences, University of Bremen, Bremen, Germany; 5 Present Address: Department of Marine Sciences, University of Gothenburg, Gothenburg, Sweden

**Keywords:** Biogeochemistry, Ecology, Ocean sciences

## Abstract

Water radiolysis continuously produces H_2_ and oxidized chemicals in wet sediment and rock. Radiolytic H_2_ has been identified as the primary electron donor (food) for microorganisms in continental aquifers kilometers below Earth’s surface. Radiolytic products may also be significant for sustaining life in subseafloor sediment and subsurface environments of other planets. However, the extent to which most subsurface ecosystems rely on radiolytic products has been poorly constrained, due to incomplete understanding of radiolytic chemical yields in natural environments. Here we show that all common marine sediment types catalyse radiolytic H_2_ production, amplifying yields by up to 27X relative to pure water. In electron equivalents, the global rate of radiolytic H_2_ production in marine sediment appears to be 1-2% of the global organic flux to the seafloor. However, most organic matter is consumed at or near the seafloor, whereas radiolytic H_2_ is produced at all sediment depths. Comparison of radiolytic H_2_ consumption rates to organic oxidation rates suggests that water radiolysis is the principal source of biologically accessible energy for microbial communities in marine sediment older than a few million years. Where water permeates similarly catalytic material on other worlds, life may also be sustained by water radiolysis.

## Abstract (web summary)

The extent to which chemical products of water radiolysis could sustain subseafloor microbial life is unknown. Here the authors show that sediment catalyzes radiolytic production of H_2_ and oxidants, providing the primary energy source for life in ancient marine sediment.

## Introduction

Radionuclides are ubiquitous in sediment and rock, where their decay leads to hydrogen (H_2_) and oxidant production via radiolysis of water^1–4^. Radiolytic yields in pure water are well constrained^5,6^ and some monominerals (pyrite, various oxides, mordenite, calcite) are known to amplify water-radiolytic H_2_ yields when irradiated by γ rays^7–9^. Similarly, other oxides and calcite enhance water-radiolytic H_2_ production following exposure to α particles^9–11^. The effect of mineralogically complex natural materials on H_2_ yields is previously unexplored.

Hydrogen (H_2_) and oxidants generated by natural radiolysis of water provide a continuous source of chemical energy for subsurface ecosystems^2–4,12,13^. Microbial life persists deep beneath Earth’s surface^14,15^ and constitutes a significant fraction of Earth’s total biomass^16,17^. Radiolytic H_2_ is now recognized as the primary electron donor for microbial communities kilometers below the surface in Precambrian regions of continental lithosphere^14^. However, the extent to which most subsurface ecosystems rely on radiolytic products has been unclear because (i) radiolytic chemical yields in natural environments have been poorly constrained and (ii) organic matter and oxidants from the surface photosynthetic world are pervasive in many subsurface environments.

## Results and discussion

We experimentally quantified H_2_ yields for α- and γ-irradiation of pure water, seawater, and seawater-saturated marine sediment with a typical abyssal clay porosity (80–85%) for all abundant marine sediment types (abyssal clay, nannofossil-bearing clay (calcareous marl), clay-bearing siliceous ooze, calcareous ooze, and lithogenous sediment), which collectively cover ~70% of Earth’s surface.

**Fig. 1:**
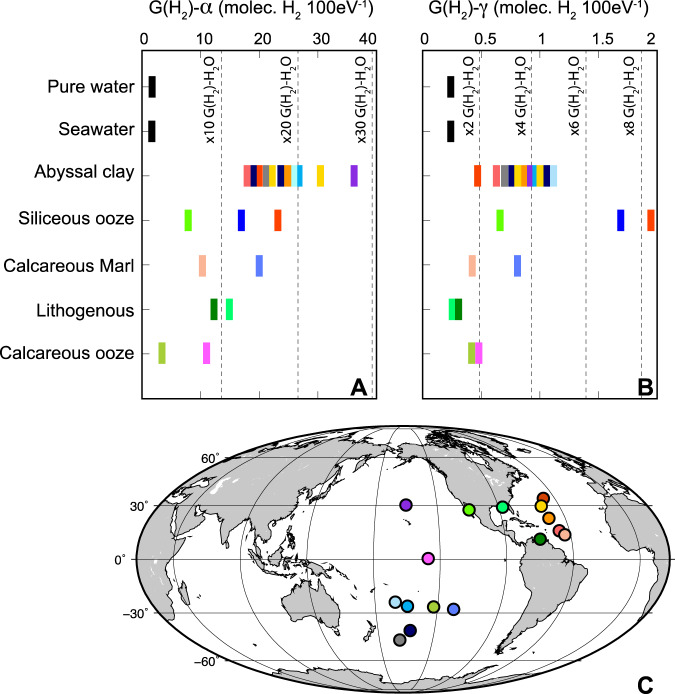
Radiolytic H_2_ catalysis by marine sediment. Experimental H_2_ yields for α irradiation (**A**) and γ irradiation (**B**). Reported yields are averages of a minimum of four replicate experiments. Vertical dashed lines represent multiples of production in pure water. Site locations (**C**) color-coded to indicate origins of samples in **A** and **B**.

Our results show that for pure water, seawater, and marine sediment slurries, H_2_ production increases linearly with absorbed α- and γ-ray dose. Energy-normalized radiolytic H_2_ yields, denoted by G(H_2_) (molecules H_2_ per 100 eV absorbed)^1^, in seawater are indistinguishable from those in pure water, within the 90% confidence limit of our experiments. In contrast, G(H_2_) values of marine sediment slurries are consistently higher than values for pure water (Fig. 1). The catalytic effect of marine sediment on radiolytic yield is significant for both α- and γ-irradiation, but much larger for α-irradiation. Alpha-irradiation G(H_2_) values for abyssal clay slurries are more than an order of magnitude higher than for pure water. On average, clay-bearing siliceous ooze and calcareous marl increase G(H_2_) for α-irradiation by factors of 15 and 12, respectively. Calcareous ooze increases yields by a factor of 5 for α-irradiation. For γ-irradiation, clay-bearing siliceous ooze and abyssal clay amplify G(H_2_) by factors of 8 and 4, respectively. Calcareous ooze and marl slurries doubled G(H_2_) for γ-irradiation. These results demonstrate that (i) all common marine sediment types catalyze radiolytic H_2_ production, and (ii) the magnitude of this catalysis depends on sediment composition and radiation type.

Previous experiments with oxides suggest that the primary cause of increased yield in all of our sediment types is energy transfer from sediment particles to the water via excitons^18–20^. H_2_ yield exceeds the pure-water yield for γ-irradiated oxides characterized by a band gap equal to the 5.1 eV energy of the H–OH bond in water^18^. This result is consistent with irradiation of the oxide generating excitons that propagate to the oxide–water interface, where they lyse the water^18–20^. With excitons as the primary mechanism for transferring irradiation energy from sediment particles to particle–water interfaces^18,19^, factors that may cause variation in radiolytic H_2_ catalysis from one sediment type to another include mineral composition of the sediment (which affects band gap), particle size (which affects exciton migration distance), water adsorption form (physisorbed vs. chemisorbed), and surface density of hydroxyl groups^20^.

In addition to H_2_, water radiolysis generates diverse oxidized products in wet sediment. In pure water, production of H_2_ from radiolysis is stoichiometrically balanced by production of H_2_O_2_ [2H_2_O → H_2_ + H_2_O_2_]^12,21^. In the presence of reduced chemicals, such as reduced sulfur and/or reduced iron, H_2_ production is balanced by production of H_2_O_2_ plus oxidation of iron and sulfur^7,22^. H_2_O_2_ spontaneously decomposes to ½O_2_ + H_2_O^23^; its rate of spontaneous decomposition is catalytically increased by common minerals^24,25^, and it readily oxidizes sulfide and iron^26^.

Many microorganisms can directly or indirectly utilize radiolytic H_2_ and/or oxidized radiolytic products as an electron donor and terminal electron acceptor, respectively^3,27^. Oxidizing power from water radiolysis both benefits and challenges microbes. Oxygen, oxidized iron, and oxidized sulfur are used as terminal electron acceptors by diverse microorganisms^3,28,29^ and some bacteria can use H_2_O_2_ directly as a terminal electron acceptor^30^. However, the H_2_O_2_, its degradation product O_2_, and the short-lived reactive oxygen species (ROS) that react to produce the H_2_O_2_, oxidized metal, and oxidized sulfur, can oxidatively stress microbes^31^. Furthermore, ROS are critical for multiple biological processes (gene expression, intracellular signaling, cell defense)^32^. In short, subseafloor microbes need to manage these chemicals (particularly short-lived ROS) but can energetically benefit from reducing relatively stable radiolytic products (including H_2_O_2_, O_2_, oxidized iron, and sulfur species).

**Fig. 2:**
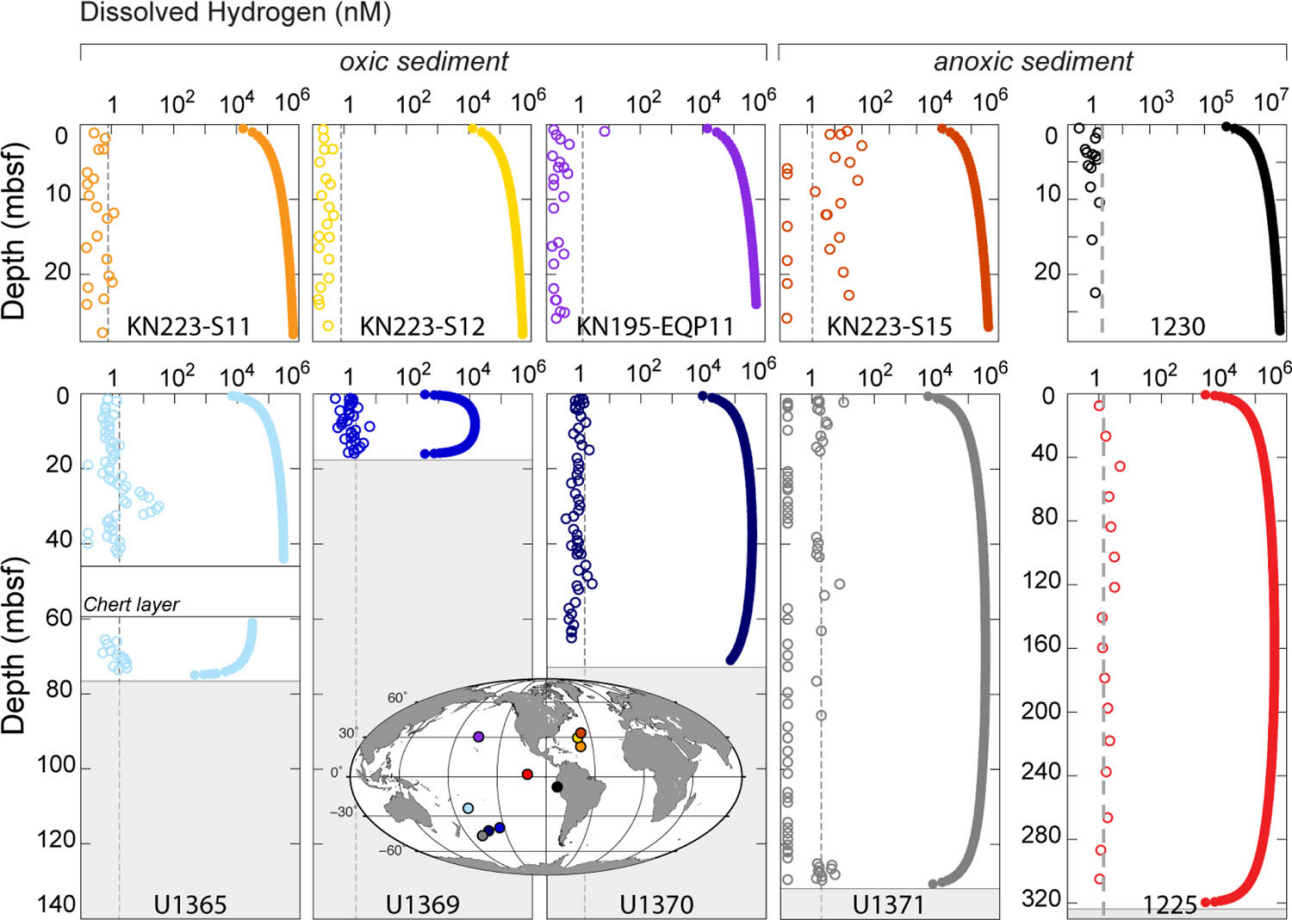
Sedimentary profiles of dissolved H_2_ concentrations. Dissolved H_2_ concentration profiles for North Pacific Site KN195-EQP11, South Pacific IODP Sites U1365, U1369, U1370, and U1371, North Atlantic Sites KN223-11, KN223-12 and KN223-15, Equatorial Pacific ODP Site 1225, and Peru Trench ODP Site 1230. Open symbols mark measured H_2_ concentrations. Solid symbols represent H_2_ concentrations expected from radiolytic H_2_ production and diffusion in the absence of in situ H_2_ consumption. Gray vertical lines mark detection limits for dissolved H_2_ concentration measurements. The detection limit of the applied analytical protocol was defined as the mean of the repeated procedural blank measurements plus three times their standard deviation. Symbol colors match site locations in Fig. 4.

Despite continual radiolytic production throughout the sediment column, dissolved H_2_ concentrations are mostly below the detection limit (which ranges from 1–5 nM H_2_ from site to site) in oxic subseafloor sediment^33,34^ and low to below detection (1–30 nM H_2_) in anoxic subseafloor sediment^35^ (Fig. 2). Measured in situ H_2_ concentrations are generally 2 to 5 orders of magnitude lower than expected from radiolytic production in the absence of H_2_-consuming reactions (Fig. 2). This discrepancy between measured and expected concentrations indicates that consumption of radiolytic H_2_ is essentially equal to its production throughout the sediment. The simplest explanation is microbial H_2_ oxidation at all depths, since enzymatic potential for H_2_ oxidation is ubiquitous in marine sediment^36^ and the in situ Gibbs energy of H_2_ oxidation is energy-yielding at the H_2_ detection limit throughout these sequences (Supplementary Information). Although this oxidation of radiolytic H_2_ contributes to gross redox activity, it does not contribute to net activity (e.g., net oxidant reduction) because water radiolysis generates oxidants in stoichiometric balance with H_2_.

**Fig. 3:**
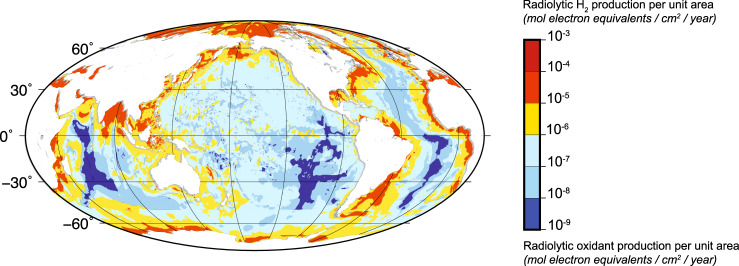
Global distribution of production rates of radiolytic H_2_ and radiolytic oxidants in marine sediment. Rates are expressed in mol electron equivalents/cm^2^/year. In electron equivalents, water radiolysis produces H_2_ and oxidized chemicals at equal rates. Although abyssal clay has a volumetric radiolytic production rate ∼ an order of magnitude higher than the volumetric rate for continental-margin sediment types, the sediment layer that blankets open-ocean regions is much thinner and consequently has much lower vertically integrated radiolytic production than the sediment of continental margins.

We assess the potential contribution of water radiolysis in marine sediment to global bioenergy fluxes by quantifying global production of radiolytic H_2_ and radiolytic oxidants in marine sediment (Fig. 3). Our estimates of radiolytic H_2_ and oxidant production are based on (i) spatial integration of a previously published model of sedimentary water radiolysis^2^, (ii) our dataset of experimentally constrained radiolytic H_2_ yields for the principal marine sediment types, and (iii) the global distribution of sediment properties (details in “Methods”). Radiolytic production of H_2_ and oxidants per unit area varies by five orders of magnitude from site to site, depending primarily on sediment column thickness (Fig. 3). Global production rates of radiolytic H_2_ and oxidants in marine sediment are 2.7 × 10^13^ mol electron equivalents per year (mol e^−^_eq_ yr^−1^).

These rates are significantly higher than a recent estimate of the radiolytic H_2_ production in Precambrian lithosphere (3.2–9.4 × 10^10^ mol e^−^_eq_ yr^−1^)^13^, which covers 72% of global continental area. Although consideration of mineral catalysis would increase the estimate for Precambrian lithosphere, it would probably not erase the difference between marine sediment and Precambrian lithosphere, because porosity is much higher and particle size is much smaller in marine sediment^2^ than in Precambrian lithosphere^13^.

Our estimated rates of radiolytic chemical production in marine sediment (2.7 × 10^13^ mol e^−^_eq_ yr^−1^) are almost two orders of magnitude lower than the flux of organic matter (organic carbon and organic nitrogen) to the seafloor (~1 × 10^15^ mol e^−^_eq_ yr^−1^)^27^ and roughly an order of magnitude lower than the burial rate of organic matter in marine sediment (0.7–3.4 × 10^14^ mol e^−^_eq_ yr^−1^)^27^. At steady state, the rate of organic consumption in marine sediment is approximated by the difference between the organic flux to the seafloor and the organic burial rate. Based on this difference, global rates of radiolytic H_2_ production and radiolytic electron acceptor production in marine sediment are only 1–2% of global organic consumption in marine sediment (in electron equivalents). However, most organic consumption in marine sediment occurs at the seafloor and organic consumption rate generally decreases exponentially with sediment depth^27^. In contrast, production of radiolytic H_2_ and radiolytic oxidants is relatively constant throughout the sediment column and not focused near the seafloor.

**Fig. 4:**
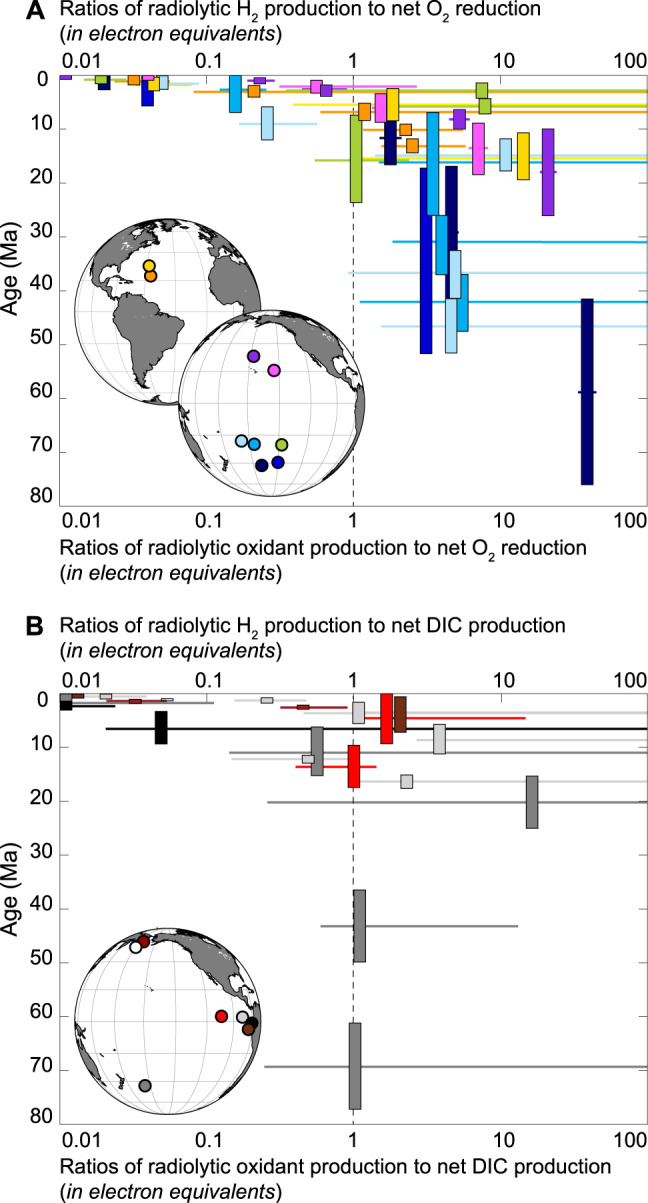
Metabolic contribution of radiolytic products in marine sediment. **A** Ratios of (i) radiolytic H_2_ production to net O_2_ reduction and (ii) radiolytic oxidant production to net O_2_ reduction, plotted against sediment age for sites with oxic deep subseafloor sediment. Horizontal lines represent one standard deviation of the ratio of radiolytic H_2_ production to net O_2_ reduction. **B** Ratios of (i) radiolytic H_2_ production to net dissolved inorganic carbon (DIC) production and (ii) radiolytic oxidant production to net DIC production for sites with anoxic deep subseafloor sediment. Horizontal lines represent one standard deviation of the ratio of radiolytic H_2_ production to net DIC production. The transition from ratios below 1 (organic oxidation dominance) in young sediment to ratios above 1 (radiolytic dominance) in older sediment results from large decreases in organic oxidation rates with increasing sediment age; radiolytic production of H_2_ and oxidants is relatively constant with sediment age, assuming no major changes in sediment composition.

We assess the potential importance of radiolytic products as an energy source for oxic subseafloor sedimentary ecosystems, by comparing production rates of radiolytic chemicals to net O_2_ reduction rates at nine sites with oxic subseafloor sediment, where organic matter concentrations are low but electron acceptors are abundant^33^. Redfield stoichiometry of dissolved NO_3_^−^ to O_2_ indicates that net O_2_ consumption in these sequences is almost entirely due to organic oxidation^33^. In sediment deposited during the last few million years, the ratio of radiolytic H_2_ production to net O_2_ consumption is generally less than 1 (Fig. 4A), indicating that microbial respiration is primarily based on oxidation of organic matter. In older sediment, this ratio is generally greater than 1, implying that radiolytic H_2_ is the primary electron donor (Fig. 4A). To the extent that oxidation of reduced metals in the sediment [e.g. Mn(II), Fe(II)] also contributes to net O_2_ consumption, this ratio overestimates the importance of organic matter as an electron donor relative to radiolytic H_2_.

To evaluate the potential role of radiolytic products for sustaining subseafloor communities in anoxic sediment, we compare radiolytic production rates to dissolved inorganic carbon (DIC) production rates for seven sites from the Pacific Ocean and Bering Sea (Fig. 4B). DIC is the primary oxidized product of organic-fueled catabolism. As with oxic sediment, in anoxic sediment younger than a few Ma, this ratio is generally less than 1.0, indicating that organic matter is the primary electron donor. However, the ratio is generally at or above above 1 in older anoxic sediment (Fig. 4B).

Collectively, these findings suggest that radiolytic H_2_ is the primary electron donor in marine sediment older than a few Ma. Given the stoichiometry of water radiolysis^12^, these results also suggest that radiolysis is the primary source of electron acceptors in marine sediment older than a few Ma (Fig. 4A, B). This continuous release of oxidants through sediment-catalyzed water radiolysis may sustain diverse redox processes in anoxic sediment, such as (i) NO_3_^−^ reduction inferred from transcriptomic signatures^37^ and (ii) SO_4_^2−^ reduction inferred from radiotracer incubations^38^ of samples taken from sediment deep beneath the last subseafloor occurrences of measurable dissolved NO_3_^−^ and SO_4_^2−^. Decomposition of radiolytic H_2_O_2_ to O_2_ is also consistent with most bacterial isolates from anoxic subseafloor sediment being facultative aerobes^39^. Comparison to continental data^13,16^ indicates that subseafloor sedimentary life constitutes one of Earth’s largest radiolysis-supported biomes.

This study reveals the importance of abundant geological materials as catalysts of radiolytic chemical production. Explicit recognition of this catalytic effect is necessary to constrain habitable zones within subsurface environments on Earth and other planetary bodies.

Although modern marine sediment typically contains biogenic components (carbonate and/or opal microfossils, fish teeth, etc.), its mineral composition directly overlaps with the mineral composition of early Earth and other planetary bodies. The known catalytic minerals abundant in marine sediment (zeolites and calcite) are inferred to have existed on early Earth^40^. Zeolites and other minerals dominant in marine sediment (smectite, chlorite, opaline silica) are present on Mars^41–43^.

On modern Earth, naturally catalyzed radiolytic products appear to provide the dominant fuel for microbial activity in marine sediment older than a few Ma. Where catalytic materials were present, radiolytic products may also have been significant for pre-photosynthetic life on early Earth and where water permeates similarly catalytic material on other planets and moons, such as the subsurface environments of Mars^42^, Europa, or Enceladus, life may similarly be sustained today.

## Methods

### Radiation experiments

We experimentally quantified radiolytic hydrogen (H_2_) production in (i) pure water, (ii) seawater, and (iii) seawater-saturated sediment. We irradiated these materials with α- or γ-radiation for fixed time intervals and then determined the concentrations of H_2_ produced. Sediment samples were slurried with natural seawater to achieve a slurry porosity (*φ*) of ~0.83, which is the average porosity of abyssal clay in the South Pacific Gyre^34^. The seawater source is described below. To avoid microbiological uptake of radiolytic H_2_ during the course of the experiment, seawater and marine sediment slurries were pre-treated with HgCl_2_ (0.05% solution) or NaN_3_ (0.1% wt/vol). To ensure that addition of these chemicals did not impact radiolytic H_2_ yields, irradiation experiments with pure water plus HgCl_2_ or NaN_3_ were also conducted. HgCl_2_ or NaN_3_ addition had no statistically significant impact on H_2_ yields^5,6,10^.

Experimental samples were irradiated in 250 mL borosilicate vials. A solid-angle ^137^Cs source (beam energy of 0.67 MeV) was used for the γ-irradiation experiments at the Rhode Island Nuclear Science Center (RINSC). The calculated dose rate for sediment slurries was 2.19E−02 Gy h^−1^ accounting for the (i) source activity, (ii) distance between the source and the samples, (iii) sample vial geometry, and (iv) attenuation coefficient of γ-radiation through air, borosilicate, and sediment slurry. ^210^Po (5.3 MeV decay^−1^)-plated silver strips with total activities of 250 μCi were used for the α-irradiation experiments. For α-irradiation of each sediment slurry, a ^210^Po-plated strip was placed inside the borosilicate vial and immersed in the slurry. Calculated total absorbed doses were 4 Gy and 3 kGy for γ-irradiation and α-irradiation experiments, respectively.

The settling time of sediment grains in the slurries (1 week) was long compared to the time span of each experiment (tens of minutes to an hour for α-experiments, hours to days for γ-experiments). Therefore, we assumed that the suspension was homogenous during the course of each experiment.

H_2_ concentrations were measured by quantitative headspace analysis via gas chromatography. For headspace analysis, 30 mL of N_2_ was first injected into the sample vial. To avoid over-pressurization of the sample during injection, an equivalent amount of water was allowed to escape through a separate needle. The vials were then vigorously shaken for 5 min to concentrate the H_2_ into the headspace. Finally, a 500-μL-headspace subsample was injected into a reduced gas analyzer (Peak Performer 1, PP1). The reduced gas analyzer was calibrated using a 1077 ppmv H_2_ primary standard (Scott-Marrin, Inc.). A gas mixer was used to dilute the H_2_ standard with N_2_ gas to obtain various H_2_ concentrations and produce a five-point linear calibration curve (0.7, 2, 5, 20, and 45 ppm). H_2_ concentrations of procedural blanks consisting of sample vials filled with non-irradiated deionized 18-MΩ water were also determined. The concentration detection limit obtained using this protocol was 0.8–1 nM H_2_. Relative error was less than 5%. Radiation experiments were performed at a minimum in triplicate.

### Sample selection and experimental radiolytic H_2_ yields, G(H_2_)

Millipore Milli-Q system water was used for our pure-water experiments. For seawater experiments, we used bottom water collected in the Hudson Canyon (water depth, 2136 m) by RV *Endeavor* expedition EN534. Salinity of North Atlantic bottom water in the vicinity of the Hudson Canyon (34.96 g kg^−1^) is similar to that of mean open-ocean bottom water (34.70 g kg^−1^)^44,45^.

The 20 sediment samples used for the experiments were collected by scientific coring expeditions in three ocean basins (expedition KN223 to the North Atlantic^46^, expedition KN195-3 to the Equatorial and North Pacific^47^, International Ocean Discovery Program (IODP) Expedition 329 to the South Pacific Gyre^34^, MONA expedition to the Guaymas Basin^48^, expedition EN32 to the Gulf of Mexico^49^, and expedition EN20 to the Venezuela Basin^50^). To capture the dominant sediment types present in the global ocean, we selected samples typical of five common sediment types [abyssal clay (11 samples), nannofossil-bearing clay or calcareous marl (2 samples), clay-bearing diatom ooze (3 samples), calcareous ooze (2 samples), and lithogenous sediment (2 samples)]. The locations, lithological descriptions, and mineral compositions of the samples are given in Supplementary Tables 1,  2,  3, and Supplementary Fig. 1. Additional chemical and physical descriptions of the sediment samples used in the radiation experiments can be found in the expedition reports for the expeditions on which the samples were collected^34,46^.

Energy-normalized radiolytic H_2_ yields are commonly expressed as G(H_2_)-values (molecules H_2_ per 100 eV absorbed)^1^. As shown in Supplementary Fig. 2, for all irradiated samples (pure water, seawater, and marine sediment slurries), H_2_ production increased linearly with absorbed α- and γ-ray-dose. We calculated G(H_2_)-values for each sample and radiation type (α or γ) as the slope of the least-square regression line of radiolytic H_2_ concentration versus absorbed dose (Supplementary Fig. 2). The error on the yields is less than 10% for each sample. G(H_2_)-values for each sample and radiation type (α or γ) are reported in Supplementary Table 3.

Although radiolytic OH• is known to react with dissolved organic matter^51^, total organic content does not appear to significantly impact radiolytic H_2_ production, since the most organic-rich sediment (e.g., Guaymas Basin and Gulf of Mexico sediment) did not yield particularly high H_2_ (Supplementary Table 3).

### Calculated radiolytic production rates of H_2_ and oxidants in the cored sediment of individual sites

We calculated radiolytic H_2_ production rates (*P*_H2_, in molecules H_2_ cm^−3^ yr^−1^) for the cored sediment column at nine sites with oxic subseafloor sediment in the North Pacific, South Pacific, and North Atlantic; and seven sites with anoxic subseafloor sediment in the Bering Sea, South Pacific, Equatorial Pacific, and Peru Margin (see Supplementary Fig. 3 for site locations). For these calculations, we used the following equation from Blair et al.^2^:

1 $$P_{{\mathrm{H}}_2} = {\sum} A _{{\mathrm{m}},i}\rho \left( {1 - {{\upvarphi}} } \right)E_i{\mathrm{G}}({\mathrm{H}}_2)_i$$

where *i* is alpha, beta, or gamma radiation; *A*_m_ is radioactivity per mass solid; φ is porosity; *ρ* is density solid; $E_i$ is decay energy; and ${\mathrm{G}}({\mathrm{H}}_2)_i$ is radiolytic yield.

We calculated radiolytic oxidant production rates for these sediment columns from the H_2_ production rates. Because H_2_ production and oxidant production are stochiometrically balanced in water radiolysis [2H_2_O → H_2_ + H_2_O_2_], the calculated radiolytic H_2_ production rates (in electron equivalents) are equal to radiolytic oxidant production rates (in electron equivalents).

The in situ γ- and α-radiation dosages in marine sediment are, respectively, 13 and 15 orders of magnitude lower than the dosage used in our experiments. Because the measured G(H_2_) for pure water in our γ-irradiation experiment (dose rate = 2.19E-02 Gy h^−1^) is statistically indistinguishable from previously published G(H_2_) values at much higher dose rates (ca. 1.00E+3 Gy h^−1^)^5^, we infer that the γ-irradiation G(H_2_) value is constant with dose rate over five orders of magnitude. Similarly, our experimental pure water H_2_ yields following α-particle irradiation from a ^210^Po-source (dose rate of 2.55E+03 Gy h^−1^) are indistinguishable from the yield obtained by Crumière et al.^6^ [G(H_2_) = 1.30 ± 0.13] for air-saturated deionized water exposed to a cyclotron-generated He^2+^ particle beam at higher dose rate (dose rate 1.62E+05 Gy h^−1^). The close similarity in H_2_ yields obtained in both experiments implies that (i) radiolytic H_2_ yield from α-particle irradiation is identical to that from cyclotron-generated He^2+^ particle irradiation, and (ii) this yield is constant over a two-orders-of-magnitude range dose rate. Therefore, we use our experimentally determined α- and γ-irradiation G(H_2_) values for the low radiation dose rate found in the subseafloor. Because the G(H_2_) of β irradiation has not been experimentally determined for water-saturated materials, we assume that the G(H_2_) of β-radiation matches the G(H_2_) of γ-radiation for the same sediment types. In pure water, their G(H_2_) values differ by only 17%^1^. Because β radiation, on average, contributes only 11% of the total radiolytic H_2_ production from the U, Th series and K decay in marine sediment, these estimates of total H_2_ production differ by only 2–5% relative to estimates where the G(H_2_) of β radiation is assumed equal to that for pure water or for α radiation of the same sediment types.

To calculate H_2_ production rates for the entire sediment column at seven South Pacific sites and two North Atlantic sites, we measured downcore sediment profiles of U, Th, and K (i) 187 sediment samples from IODP Expedition 329 Sites U1365, U1366, U1367, U1368, U1369, U1370, and U1371^34,52^, and (ii) 40 samples from KN223 expedition Sites 11 and 12 (ref. ^46^). Total U and Th (ppm) and K_2_O (wt%) for these sites are reported in the EarthChem SedDB data repository. We measured U, Th, and K abundances using standard atomic emission and mass spectrometry techniques (i.e. ICP-ES and ICP-MS) in the Analytical Geochemistry Facilities at Boston University. Sample preparation, analytical protocol, and data are reported in Dunlea et al.^52^. The precision for each element is ~2% of the measured value, based on three separate digestions of a homogenized in-house standard of deep-sea sediment.

To calculate H_2_ production rates for the sediment columns at North Pacific coring Sites EQP10 and EQP11 (ref. ^47^), we used radioactive element content data from Kyte et al.^53^, who measured chemical concentrations at high resolution in bulk sediment in core LL44-GPC3. Because Site EQP11 was cored at the same location as LL44-GPC3 (ref. ^53^) and the sediment retrieved at all three sites is homogeneous abyssal clay, we assume the radioactive element abundances measured in core LL44-GPC3 to be representative of Sites EQP10 and EQP11 (ref. ^47^). Calculated radiolytic H_2_ production rates for South Pacific sites are listed in Supplementary Table 4 and for North Atlantic and North Pacific sites in Supplementary Table 5.

For Bering Sea Sites U1343 and U1345 (ref. ^54^), sedimentary U, Th, and K content measurements are unavailable. Since sediment recovered at these two sites is primarily siliciclastic with a varying amount of diatom-rich clay, we use U, Th, and K concentration values reported for upper continental crust by Li and Schoonmaker for these Bering Sea sites^55^. Finally, we calculate downhole radiolytic H_2_ production rates for ODP Leg 201 Sites 1225, 1226, 1227, and 1230 (ref. ^35^). Sediment compositions for these sites include nannofossil-rich calcareous ooze (Site 1225), alternation of nannofossil (calcareous) ooze and diatom ooze (Site 1226), and siliciclastic with diatom-rich clay intervals (Sites 1227 and 1230). Because sedimentary U, Th, and K measurements are not available for Leg 201 sites, we used average U, Th, and K concentration values measured in North Atlantic^46^ and South Pacific Sites^34,52^ with corresponding lithologies.

We use isotopic abundance values reported in Erlank et al.^56^ to calculate the abundance of ^238^U, ^235^U, ^232^Th, and ^40^K from the measured ICP-MS values of total U, Th, and K concentration. We then converted radionuclide concentrations to activities using Avogadro’s number and each isotope’s decay constant^2^. We refer to Blair et al. for a detailed explanation of activities and radiolytic yield calculations^2^.

### Calculation of global radiolytic H_2_ and oxidant production rates in marine sediment

We calculated global radiolytic H_2_ production in ocean sediment by applying Eq. (1) (ref. ^2^) globally. As with the rates at individual sites, we calculated global radiolytic oxidant production (in electron equivalents) from global H_2_ production and the stochiometry of water radiolysis [2H_2_O → H_2_ + H_2_O_2_].

Our global radiolytic H_2_ production calculation spatially integrates calculations of sedimentary porewater radiolysis rates that are based on (i) our experimentally constrained radiolytic H_2_ yields for the principal marine sediment types, (ii) measured radioactive element content of sediment cores in three ocean basins (North Atlantic^46^, North Pacific^53^, and South Pacific^34,52^), and (iii) global distributions of sediment lithology^57^, sediment porosity^58^, and sediment column length^59,60^.

To generate the global map of radiolytic H_2_ production, we created global maps of seafloor U, Th, and K concentrations, density, G(H_2_)-α values, and G(H_2_)-γ-and-β by assigning each grid cell in our compiled seafloor lithology map (Supplementary Fig. 4) its lithology-specific set of input variables (Supplementary Table 6). Because our model assumes that lithology is constant with depth, U, Th, and K content, grain density, and G(H_2_)-values are constant with depth.

The G(H_2_)-values (α, β, and γ radiation), radioactive element content (sedimentary U, Th, and K concentration), density, porosity, and sediment thickness are determined as follows.

### Radiolytic yield [G(H_2_)] for α,β-&-γ radiation

Radiolytic yields for the main seafloor lithologies are obtained by averaging experimentally derived yields for the respective lithologies (Supplementary Table 6). We assume that G(H_2_)-β values equal G(H_2_)-γ values.

### Sediment lithology

For these calculations of radiolytic chemical production, we generally used seafloor lithologies and assumed that sediment type is constant with sediment depth. For seafloor lithology, the geographic database of global bottom sediment types^57^ was compiled into five lithologic categories: abyssal clay, calcareous ooze, siliceous ooze, calcareous marl, and lithogenous (Supplementary Fig. 4). Some areas of the seafloor are not described in the database^57^. These include (i) high-latitude regions (as the seafloor lithology database extends from 70°N to 50°S)^57^ and (ii) some discrete areas located along continental margins (e.g., Mediterranean Sea, Timor Sea, South China Sea, Supplementary Fig. 4). We used other data sources to identify seafloor lithologies for these regions. We added an opal belt (siliceous ooze) in the Southern Ocean between 57°S and 66°S^61,62^. The geographic extent of this opal belt was based on DeMaster^62^ and Dutkiewicz et al.^61^. We defined the areas of the seafloor from 50°S to 57°S, from 66°S to 90°S, and in the Arctic Ocean as mostly composed of lithogenous material, based on (i) drillsite lithologies in the Southern Ocean [ODP: Site 695 (ref. ^63^), Site 694 (ref. ^63^), Site 1165 (ref. ^64^), Site 739 (ref. ^65^)], the Bering Sea and Arctic Ocean [International Ocean Discovery Program (IODP): Sites U1343 and U1345 (ref. ^54^), Site M0002 (ref. ^66^), ODP: Site 910 (ref. ^67^), Site 645 (ref. ^68^)] and between 50°S and 57°S [Deep Sea Drilling Project (DSDP): Site 326 (ref. ^69^), Ocean Drilling Program (ODP): Site 1138 (ref. ^70^), Site 1121 (ref. ^71^)], and Dutkiewicz et al.^61^.

In the North and South Atlantic, sediment type can be very different at depth than at the seafloor. For these regions, we departed from our assumption that sediment lithology is the same at depth as at the seafloor. Subseafloor lithologies at ODP Sites [1063 (ref. ^72^), 951 (ref. ^73^), 925 (ref. ^74^), and 662 (ref. ^75^)] and IODP Sites [U1403 (ref. ^76^) and U1312 (ref. ^77^)] indicate that sediment in the Atlantic Ocean basin is generally 30–90% biogenic carbonate content and detrital clay^78^, even where the seafloor lithology is abyssal clay^57^. Therefore, regions in the Atlantic Ocean described as abyssal clay in the seafloor lithology database^57^ were characterized as calcareous marl for our calculations (Supplementary Fig. 4). Because abyssal clay catalyzes radiolytic H_2_ production at a higher rate than calcareous marl, this characterization may underestimate production of radiolytic H_2_ and radiolytic oxidants in these Atlantic regions.

### Radioactive element content

For four of the five lithologic types in our global maps (abyssal clay, siliceous ooze, calcareous ooze, and calcareous marl), we average U, Th, and K concentrations from sites in the North Atlantic^46^, North Pacific^53^, and South Pacific^34,52^. The average U, Th, and K concentration values are consistent with data reported in Li and Schoonmaker^55^ for the characteristic U, Th, and K content found in abyssal clay and calcareous ooze. For lithogenous sediment, we use U, Th, and K concentration values reported for upper continental crust by Li and Schoonmaker^55^. Lithology-specific radioactive element values are given in Supplementary Table 6 and used to calculate *A*_m,*i*_ in Eq. (1).

### Density

Characteristic density values for calcite, quartz, terrigenous clay, and opal-rich sediment were extracted from the Proceedings of the Integrated Ocean Drilling Program Volume 320/321 and are assigned to calcareous ooze, lithogenous sediment, abyssal clay, and siliceous ooze, respectively^79^.

### Global porosity

For global porosity, we use a seafloor porosity data set by Martin et al.^58^ and accounted for sediment compaction with depth by using separate sediment compaction length scales for continental-shelf (0–200 m water depth; *c*_0_ = 0.5 × 10^−3^), continental-margin (200–2500 m; *c*_0_ = 1.7 × 10^−3^), and abyssal sediment (>3500 m; *c*_0_ = 0.85 × 10^−3^)^80,81^. Once the porosity was 0.1%, the depth integration was halted.

### Global sediment thickness

We calculated global depth-integrated radiolytic H_2_ production by summing the seafloor production rates over sediment depth in one-meter intervals (Fig. 3 in main text). Sediment thickness is from Whittaker et al., supplemented with Laske and Masters where needed^82,83^.

### Ocean depth

For porosity calculations, water depths were determined using the General Bathymetric Chart of the Oceans^84^, resampled to a 5-arc minute grid, i.e. the resolution of the Naval Oceanographic Office’s Bottom Sediment Type (BTS) database “Enhanced dataset”^57^.

### Dissolved H_2_ concentration profiles

H_2_ concentrations from South Pacific Sites U1365, U1369, U1370, and U1371, and the measurement protocol, are described in ref. ^1^. H_2_ concentrations from North Atlantic KN223 Sites 11, 12, and 15, and North Pacific Site EQP11 were determined using the same protocol and are posted on SedDB (see “Data availability”). The detection limit for H_2_ ranged between 1 and 5 nM H_2_, depending on site, and is displayed as gray vertical lines in Fig. 2 of the main text. H_2_ concentrations for Equatorial Pacific Site 1225 and Peru Trench Site 1230 were measured by the “headspace equilibration technique”, which measures steady-state H_2_ levels reached following laboratory incubation of the sediment samples^85,86^.

For comparison to these measured H_2_ concentrations, we use diffusion-reaction calculations to quantify what in situ H_2_ concentrations would be in the absence of H_2_-consuming reactions. The results of these calculations are represented as solid circles (•) in Fig. 2 of the main text. Temporal changes in H_2_ concentration due to diffusive processes and radiolytic H_2_ production in situ are expressed by Eq. (2):

2 $$\frac{{\partial {\mathrm{H}}_2(x,t)}}{{\partial t}} = \frac{D}{{\varphi F}}\frac{{\partial ^2{\mathrm{H}}_2(x,t)}}{{\partial x^2}} + P(x)$$

with

*D*: the diffusion coefficient of H_2_(aq) at in situ temperature

$$\varphi$$: porosity

*F*: formation factor

*x*: depth

*Z*: sediment column thickness

$${\mathrm{H}}_2$$: hydrogen concentration

*P*: radiolytic H_2_ production rate

*t* : time.

With constant radiolytic H_2_ production, *P*(*x*) = *P* with depth,

and at steady-state,

3 $$\frac{{\partial ^2{\mathrm{H}}_2(x)}}{{\partial x^2}} = - \frac{{P\varphi F}}{D}.$$

We integrate Eq. (3) over the length *x* twice,

4 $${\mathrm{H}}_2(x) = - \frac{1}{2}\frac{{P\varphi F}}{D}x^2 + Ax + B$$

where *A* and *B* in Eq. (4) are constants of integration. We use two boundary conditions to derive the value of these constants.

Boundary condition 1: concentration of H_2_ at the sediment-water interface, *x* = 0, is zero due to diffusive loss to the overlying water column.

Boundary condition 2: concentration of H_2_ at the basement–sediment-water interface, *x* = *Z*, is zero due to diffusive loss to the underlying basement.

With these boundary conditions, $A = \frac{1}{2}\frac{{P\varphi F}}{D}Z$ and *B* = 0

and

5 $${\mathrm{H}}_2(x) = \frac{1}{2}\frac{{P\varphi F}}{D}(xZ - x^2).$$

In cases where we expect radiolytic H_2_ production rates to significantly vary with depth due to changes in lithology, we adapted the boundary conditions and applied a two-layer diffusion model to account for this variation.

### Calculation of Gibbs Energies for the Knallgas reaction

For H_2_ concentrations above the detection limits at South Pacific IODP Expedition 329 sites (Supplementary Fig. 5)^34^, we quantified in situ Gibbs energies (Δ*G*_r_) of the *Knallgas* reaction (H_2_ + ½O_2_ → H_2_O). In situ Δ*G*_r_ values depend on pressure (*P*), temperature (*T*), ionic strength, and chemical concentrations, all of which are explicitly accounted for in our calculations:

6 $$\Delta G_{\mathrm{r}} = \Delta G^\circ _{\mathrm{r}}\left( {T,P} \right) + 2.3\,RT\,{\mathrm{log}}_{10}Q$$

where:

Δ*G*_r_: in situ Gibbs energy of reaction (kJ mol H_2_^−1^)

Δ*G*°_r_(*T*,*P*): Gibbs energy of reaction under in situ *T* and *P* conditions (kJ mol H_2_^−1^)

*R*: gas constant (8.314 kJ^−1^ mol K^−1^)

*Q*: activity quotient of compounds involved in the reaction.

We use the measured composition of the sedimentary pore fluid to determine values of *Q*.

For a more complete overview of in situ Gibbs energy-of-reaction calculations in subseafloor sediment, see Wang et al.^87^.

### Calculation of organic oxidation rates (net rates of O_2_ reduction and DIC production)

We calculated the vertical distribution of net O_2_ reduction rates at nine sites where the sediment is oxic from seafloor to basement and the vertical distribution of DIC production rates at seven sites where the subseafloor sediment is anoxic (see Supplementary Fig. 3 for site locations). Dissolved O_2_ concentrations are from Røy et al.^47^ and D’Hondt et al.^88^. DIC concentrations are from ODP Leg 201 (ref. ^35^), and the Proceedings of the IODP Expedition 323 (Sites U12343, U1345)^54^ and IODP Expedition 329 (Site U1371 (ref. ^34^)).

The net rates are calculated using the MatLab program and numerical procedures of Wang et al.^89^, modified by using an Akima spline, rather than a 5-point running mean, to generate a best-fit line to the chemical concentration data. Details of the calculation protocol for O_2_ production rates and DIC production rates are respectively described in the supplementary information of D’Hondt et al.^88^ and in Walsh et al.^90^. The DIC reaction rates and their first standard deviations calculated for the seven sites are given in Supplementary Table 7.

To facilitate comparisons of radiolytic chemical rates to net DIC production rates, rates are converted to electron equivalents (2 electrons per H_2_, 4 electrons per O_2_, 4 electrons per organic C oxidized).

### Estimation of sediment ages

We estimated sediment ages for Sites U1343 and U1343 using the sediment-age model of Takahashi et al.^54^, which is based on biostratigraphic and magnetostratigraphic data. Because detailed chronostratigraphic data are not available for the remaining sites (Equatorial Pacific sites (1225 and 1226), Peru Trench Site 1230 and Peru Basin Site 1231, South Pacific sites U1365, U1366, U1367, U1369, U1370, and U1371, North Pacific sites EQP9 and EQP10, and North Atlantic sites KN223-11 and KN223-12), we used the mean sediment accumulation rate for each of these sites (Supplementary Fig. 3) to convert its sediment depth (in meters below seafloor) to sediment age (in millions of years, Ma). Mean sediment accumulation rate was calculated by dividing sediment thickness by basement age^91^ (Supplementary Table 8). For Sites 1225, 1226, 1230, 1231, U1365, U1366, U1367, U1369, U1370, and U1371, sediment thickness was determined by drilling to basement^34,35^. For Sites EQP9, EQP10, KN223-11, and KN223-12, sediment thicknesses were determined from acoustic basement reflection data.

## Supplementary information

Supplementary Information

## Data Availability

Sedimentary radioactive element content datasets [U (ppm); Th (ppm); K (reported as wt% K_2_O)] can be retrieved from the EarthChem SedDB data repository (10.1594/IEDA/100603). Code and datasets for the global estimate of radiolytic H_2_ production are available at https://code.usgs.gov/vsc/publications/flinders_a/H2-seafloor-estimator.
