# Supplementary material for: The contribution of water radiolysis to marine sedimentary life
Source: Nat Commun. 2021 Feb 26;12:1297. doi: 10.1038/s41467-021-21218-z (PMC7910440; doi:10.1038/s41467-021-21218-z)
Supplement: Supplementary file 1 — Supplementary Information [file 41467_2021_21218_MOESM1_ESM.pdf]

The contribution of water radiolysis to marine sedimentary life

Supplementary information

Justine F. Sauvage[1+*], Ashton Flinders[2], Arthur J. Spivack[1], Robert Pockalny[1], Ann G. Dunlea[3#], Chloe H. Anderson[3=], David C. Smith[1], Richard W. Murray[3#], and Steven D'Hondt[1]

[1] *Graduate School of Oceanography, University of Rhode Island, Narragansett, RI 02882, USA*

[2] *United States Geological Survey, Hawaiian Volcano Observatory, Hilo, HI*

[3] *Department of Earth and Environment, Boston University, Boston, MA 02215, USA*

[+] *now at Department of Marine Sciences University of Gothenburg, Gothenburg, Sweden.*

[#] *now at Woods Hole Oceanographic Institution, Woods Hole, MA 02543, USA*

[=] *now at Center for Marine Environmental Sciences, University of Bremen, Bremen, Germany*

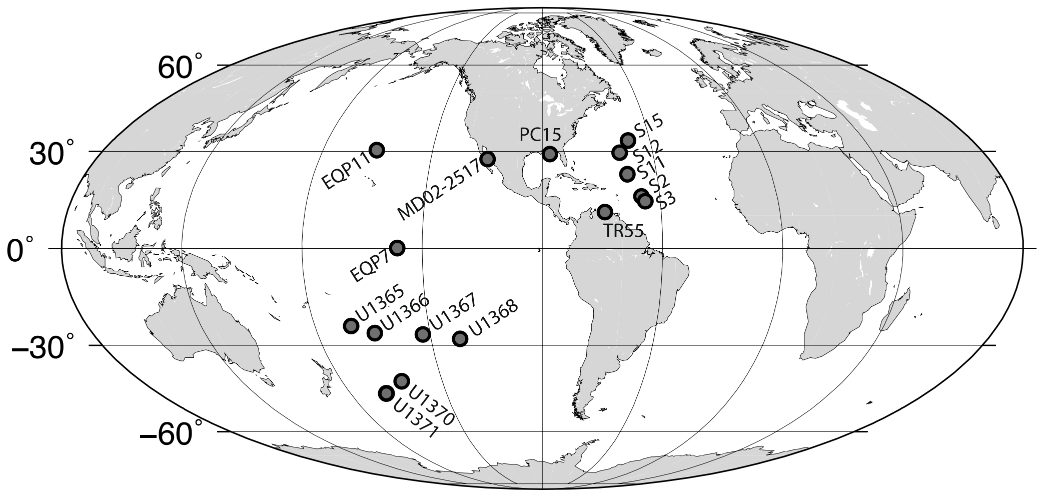

Supp. Figure 1. Site locations for the sediment samples used in the radiation experiments.

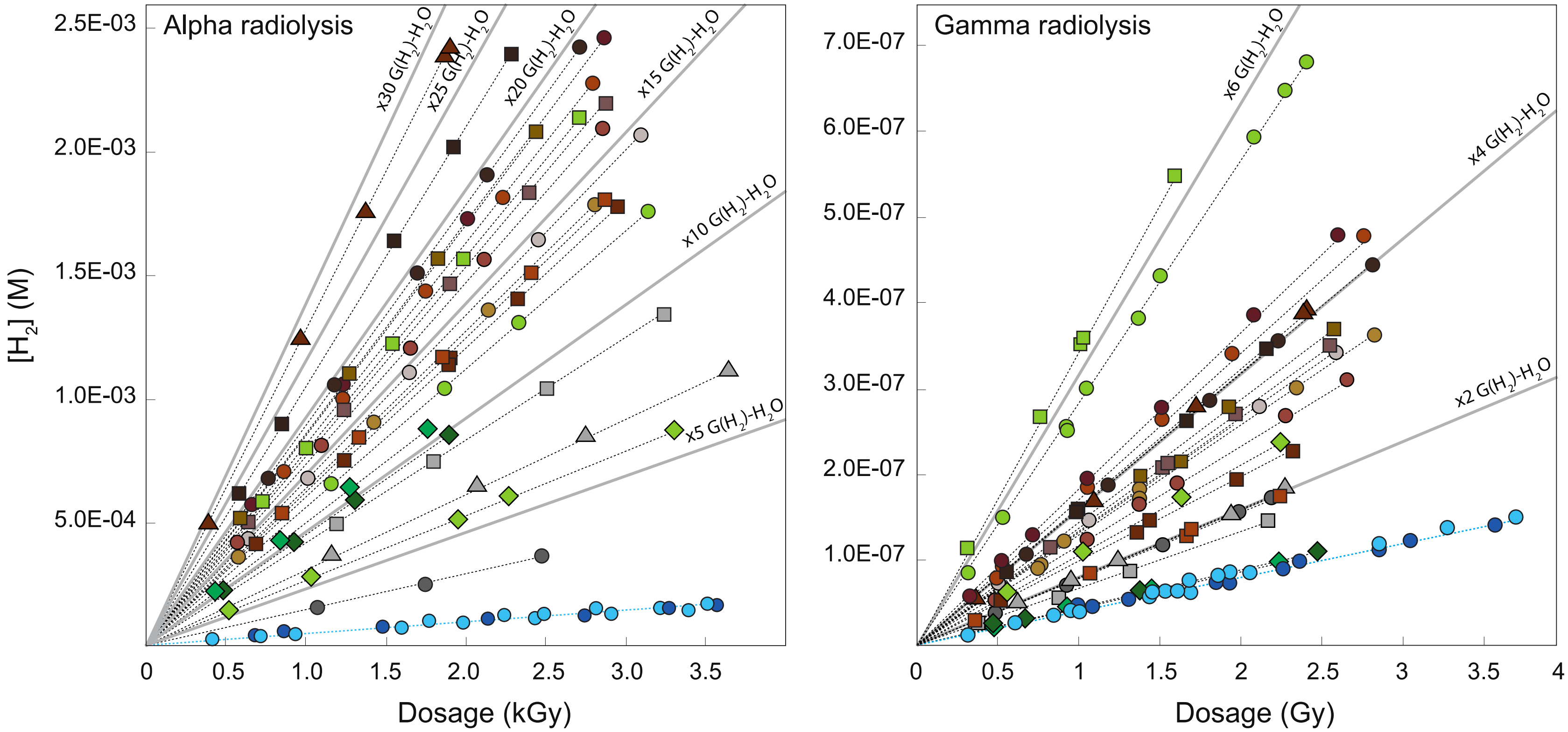

Supp. Figure 2. Production of radiolytic H_2 as a function of adsorbed dose for pure water, seawater, and marine sediment slurries irradiated by α-particles (left) and γ-rays (right). Symbols and colors match the sample information in Supplemental Tables 1 and 3. Gray solid lines represent multiples of production in pure water.

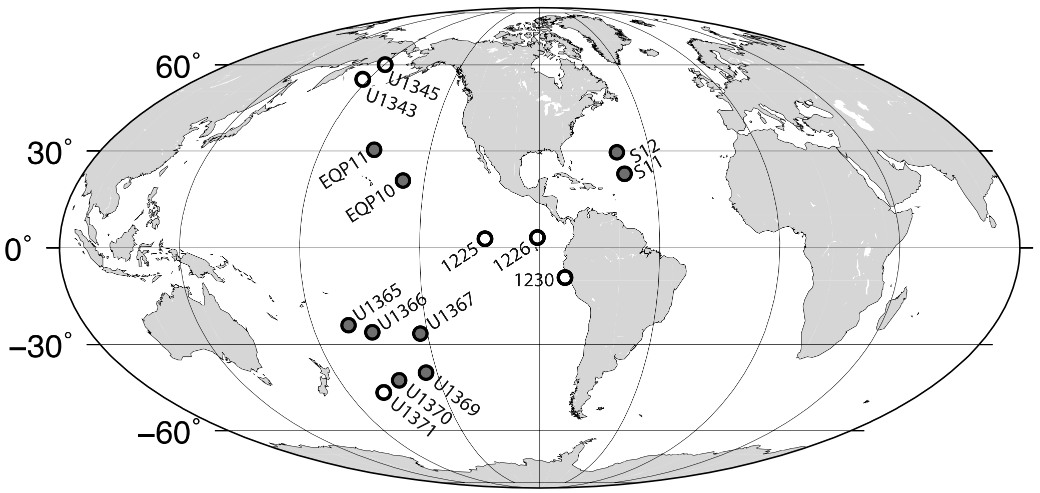

Supp. Figure 3. Sites for which we calculated radiolytic chemical production in the cored sediment column. Gray dots mark sites with oxic subseafloor sediment and white dots mark sites with anoxic subseafloor sediment.

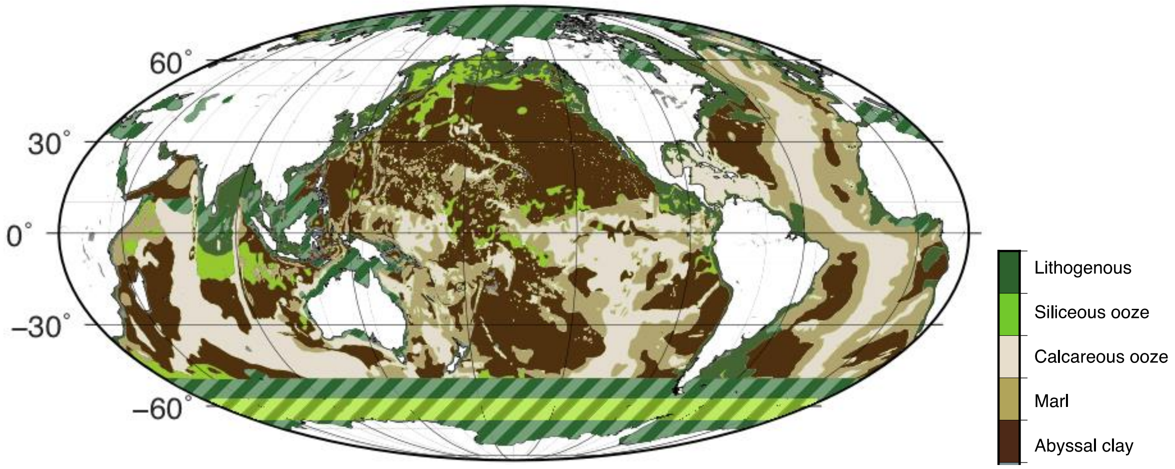

Supp. Figure 4. Geographic distributions of seafloor lithologies. Open-ocean lithologies from 70°N to 50°S are from reference 57. regions marked with a diagonal pattern (poleward of 70°N and 50°S, and near some continental margins) are not included in reference 57. Lithologies in those regions are based on drill cores within the regions (see Methods for explanation).

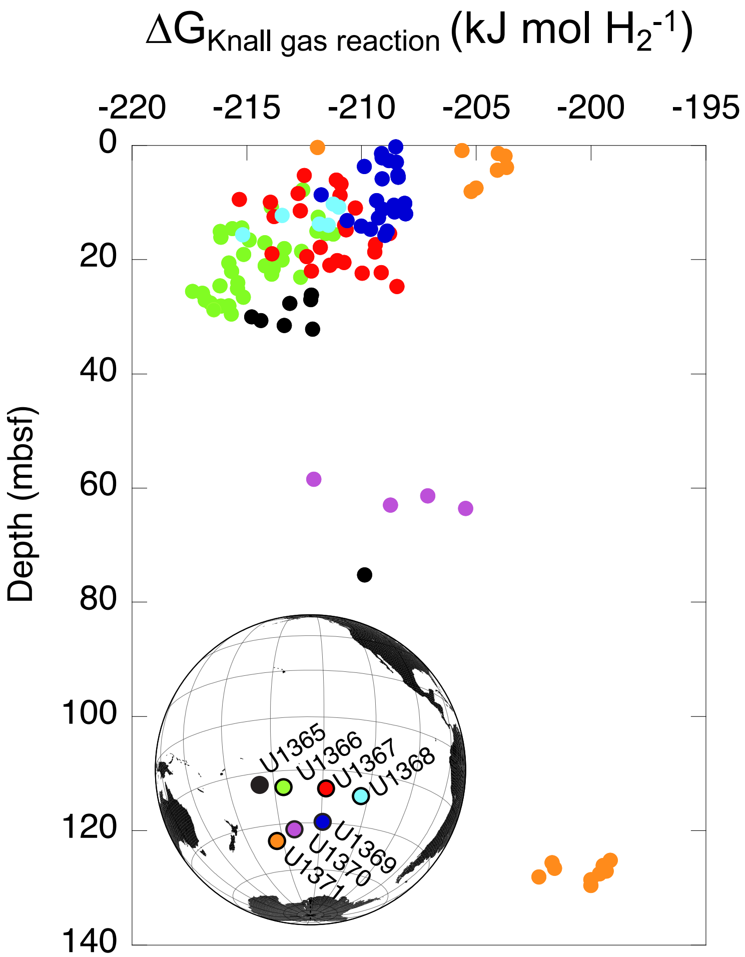

Supp. Figure 5. *In situ* Gibbs energies for the *Knallgas* reaction at IODP Expedition 329 sites (calculated for samples with H_2 concentrations above the detection limit).

Aqueous solution	Origin
Pure water	Rhode Island Nuclear Science Center - University of Rhode Island
Seawater	Hudson Canyon, USA

Lithology	Expedition	Site	Latitude	Longitude	Hole	Core	Section	Depth (mbsf)	TOC (%)
Zeolitic pelagic clay	IODP Exp. 329	U1365	23°51.05'S	165°38.66'W	B	2	5	10.8	0.07
Zeolitic pelagic clay	IODP Exp. 329	U1366	26°03.10'S	156°53.66'W	D	2	4	13.9	0.06
Zeolitic pelagic clay	IODP Exp. 329	U1370	41°51.13'S	153°06.38'W	E	2	4	11.2	0.03
Zeolitic pelagic clay	IODP Exp. 329	U1370	41°51.13'S	153°06.38'W	D	7	4	58.4	0.02
Zeolitic pelagic clay	IODP Exp. 329	U1371	45°57.85'S	163°11.05'W	E	13	1	112.5	0.04
Pelagic clay	KN223	site 2	15°50.90'N	52°00.00'W	n.a.	LC1	5	6.2	0.04
Pelagic clay	KN223	site 11	22°47.08'N	56°31.06'W	n.a.	LC1	7	8.8	0.04
Pelagic clay	KN223	site 12	29°40.60'N	58°19.70'W	n.a.	LC1	7	8.1	0.06
Pelagic clay	KN223	site 12	29°40.60'N	58°19.70'W	n.a.	LC1	13	17.2	0.08
Pelagic clay	KN223	site 15	33°29.01'N	54°09.98'W	n.a.	LC1	2	2.9	0.06
Pelagic clay	KN195	EQP11	30°21.29'N	157°52.26'W	n.a.	LC1	n.a.	28.6	0.07
Clay-bearing diatom ooze	IODP Exp. 329	U1371	45°57.85'S	163°11.05'W	E	4	1	28.5	0.01
Siliceous (radiolarian) clay	KN223	site 15	33°29.01'N	54°09.98'W	n.a.	LC1	18	26.4	0.07
Clay-bearing diatom ooze	MONA	MD02-2517	27°29.10'N	112°04.46'W	n.a.	n.a.	n.a.	0.6	0.17
Clay-bearing nannofossil ooze	IODP Exp. 329	U1368	27°55.01'S	123°09.66'W	C	2	2	9.7	0.06
Clay-bearing nannofossil ooze	KN223	site 3	14°24.04'N	50°37.27'W	n.a.	LC1	18	26.8	0.32
Calcareous ooze	IODP Exp. 329	U1367	26°28.90'S	137°56.36'W	C	2	1	7.9	0.07
Calcareous ooze	KN195	site 7	00°00.32'N	139°18.92'W	n.a.	LC1	n.a.	0.3	0.04
Lithogenous	EN32	PC15	29°10.10'N	86°59.90'W	n.a.	1	1	0.9	1.18
Lithogenous	EN20	TR55	10°39.01'N	65°04.59'W	n.a.	1	1	0.6	5.76

Supp. Table 1. Sampling locations and descriptions of the water and sediment samples used in our α and γ radiation experiments (n.a. = not available, TOC = total organic content).

Ocean Basin	Site	Depth (mbsf)	Mineral content
South Pacific Source: 34	IODP Site U1365	10.8	Clay (smectite, chlorite), zeolite, red-brown to yellow-brown semi-opaque oxide ("RSO")
	IODP Site U1366	13.9	RSO, clay (smectite, chlorite), zeolite (phillipsite)
	IODP Site U1367	7.9	Calcite, RSO, clay
	IODP Site U1368	9.7	Calcite, RSO, clay (smectite)
	IODP Site U1370	11.2	RSO, zeolite (phillipsite), clay (illite-smectite)
		58.4	RSO, zeolite (phillipsite), clay (illite-smectite)
	IODP Site U1371	28.5	Biogenic opal, volcanic glass, clay (illite, chlorite), quartz
		112.5	Clay (illite-smectite), zeolite (phillipsite), volcanic glass, RSO, manganese oxide/oxyhydroxide
North Atlantic Source: 46	KN223 Site 2	6.2	Clay, manganese oxide
	KN223 Site 3	26.8	Calcite, clay manganese oxide
	KN223 Site 11	8.8	Clay, biogenic opal, quartz, plagioclase
	KN223 Site 12	8.1	Clay
		17.2	Clay
	KN223 Site 15	2.9	Clay, calcite
		26.4	Clay, biogenic opal
North Pacific	KN195-EQP7	0.3	Calcite, biogenic opal, clay, manganese oxide
	KN195-EQP11	28.6	Clay, manganese oxide, volcanic ash

Supp. Table 2. Mineral contents of sediment sampled for our α and γ radiation experiments. Mineral contents for the North Atlantic and North Pacific sites are from unpublished shipboard core descriptions. These data are generally limited to major components and broad mineral categories.

Aqueous solution			$G(H_2)$-g	$G(H_2)$-α
Pure water			0.24	1.38
seawater			0.25	1.32
**Sediment**	**Expedition**	**Site**	**$G(H_2)$-g**	**$G(H_2)$-α**
Zeolitic pelagic clay	IODP Exp. 329	U1365	1.10	26.22
Zeolitic pelagic clay	IODP Exp. 329	U1366	0.95	28.15
Zeolitic pelagic clay	IODP Exp. 329	U1370	1.04	24.36
Zeolitic pelagic clay	IODP Exp. 329	U1370	0.78	19.32
Zeolitic pelagic clay	IODP Exp. 329	U1371	0.70	21.93
Pelagic clay	KN223	site 2	0.63	17.94
Pelagic clay	KN223	site 11	0.86	25.53
Pelagic clay	KN223	site 12	0.83	22.22
Pelagic clay	KN223	site 12	0.95	31.47
Pelagic clay	KN223	site 15	0.47	18.63
Pelagic clay	KN195	EQP11	0.99	37.54
Clay-bearing diatom ooze	IODP Exp. 329	U1371	1.69	16.97
Siliceous (radiolarian) clay	KN223	site 15	1.96	23.18
Clay-bearing diatom ooze	MONA	MD02-2517	0.66	7.87
Clay-bearing nannofossil ooze	IODP Exp. 329	U1368	0.81	20.01
Clay-bearing nannofossil ooze	KN223	site 3	0.43	11.45
Calcareous ooze	IODP Exp. 329	U1367	0.47	3.43
Calcareous ooze	KN195	EQP7	0.48	8.97
Lithogenous	EN32	PC15	0.27	15.56
Lithogenous	EN20	TR55	0.29	13.65

Supp. Table 3. $G(H_2)$-values (expressed in molecules H_2/100eV) for each sample and radiation type (γ and α).

South Pacific									
U1365		U1366		U1367		U1369		U1370	
**Depth (mbsf)**	**H_2 yield (mol H_2/ yr/cm^3_{sed})**	**Depth (mbsf)**	**H_2 yield (mol H_2/ yr/cm^3_{sed})**	**Depth (mbsf)**	**H_2 yield (mol H_2/ yr/cm^3_{sed})**	**Depth (mbsf)**	**H_2 yield (mol H_2/ yr/cm^3_{sed})**	**Depth (mbsf)**	**H_2 yield (mol H_2/ yr/cm^3_{sed})**
0.5	1.06E-11	0.35	1.83E-11	0.05	2.31E-11	0.1	1.57E-11	0.1	1.36E-11
0.35	1.06E-11	1.35	1.93E-11	0.55	2.38E-11	0.45	1.49E-11	0.475	1.40E-11
0.85	1.17E-11	2.05	1.93E-11	1.05	2.45E-11	1.45	1.56E-11	1.45	1.31E-11
1.35	1.15E-11	2.35	1.97E-11	3.05	2.54E-11	2.45	1.39E-11	2.45	1.38E-11
3.75	1.26E-11	3.35	1.84E-11	4.45	2.55E-11	2.95	1.37E-11	3.45	1.44E-11
5.45	6.89E-12	4.35	1.86E-11	5.05	2.62E-11	3.45	1.32E-11	7.65	1.49E-11
6.30	6.97E-12	4.85	1.88E-11	6.05	4.17E-13	3.95	1.50E-11	9.15	1.55E-11
7.80	1.22E-11	5.35	1.87E-11	6.55	6.02E-13	4.45	1.44E-11	12.15	1.97E-11
8.45	1.42E-11	5.85	1.80E-11	7.12	3.66E-13	5.77	1.55E-11	13.65	1.82E-11
9.95	2.73E-11	6.35	1.79E-11	8.25	1.03E-12	7.45	2.49E-11	14.88	1.95E-11
11.45	2.63E-11	6.85	1.65E-11	8.65	8.97E-13	8.95	2.65E-11	17.15	1.90E-11
13.63	1.87E-11	7.35	1.61E-11	9.25	1.59E-13	10.45	2.14E-11	18.65	1.72E-11
15.80	2.23E-11	8.35	1.49E-11	9.75	1.90E-13	11.95	2.01E-11	20.15	2.03E-11
17.95	2.17E-11	8.85	2.09E-11	12.75	9.41E-13	12.95	2.18E-11	21.65	1.87E-11
21.80	1.20E-11	9.75	1.76E-11	15.25	4.50E-12	13.95	2.27E-11	23.15	1.89E-11
24.45	1.30E-11	10.25	1.83E-11	18.25	6.32E-12			26.65	1.96E-11
25.30	1.21E-11	10.75	2.20E-11					29.65	2.38E-11
28.30	1.21E-11	11.75	2.24E-11					32.65	2.28E-11
31.95	1.33E-11	12.25	2.53E-11					36.15	2.56E-11
33.95	1.27E-11	12.75	2.69E-11					39.65	2.50E-11
35.45	1.15E-11	13.75	2.56E-11					42.79	2.55E-11
35.65	1.19E-11	14.76	2.56E-11					47.09	2.37E-11
37.80	1.03E-11	15.00	2.56E-11					51.18	2.82E-11
40.80	5.91E-12	15.25	2.65E-11					55.65	2.59E-11
42.18	6.58E-12							57.15	3.36E-11
65.35	2.83E-12							58.65	4.99E-11
68.85	6.08E-12							60.15	2.84E-11
69.35	6.19E-12							61.65	2.94E-11
70.90	8.38E-12							63.15	2.02E-11
72.90	7.21E-12							63.55	1.14E-11
73.9	9.03E-12							65.05	1.55E-11
74.75	1.05E-11								

Supp. Table 4. Calculated radiolytic H_2 production rates at South Pacific sites. mbsf = meters beneath the seafloor.

North Pacific				North Atlantic			
EQP 10		EQP 11		Site 11		Site 12	
**Depth (mbsf)**	**H_2 yield (mol H_2/ yr/cm^3_{sed})**	**Depth (mbsf)**	**H_2 yield (mol H_2/ yr/cm^3_{sed})**	**Depth (mbsf)**	**H_2 yield (mol H_2/ yr/cm^3_{sed})**	**Depth (mbsf)**	**H_2 yield (mol H_2/ yr/cm^3_{sed})**
0.25	5.30E-11	0.25	5.30E-11	0.03	8.08E-11	0.03	3.00E-11
0.57	4.96E-11	0.57	4.96E-11	0.40	8.08E-11	0.09	3.12E-11
0.71	5.01E-11	0.71	5.01E-11	0.42	3.41E-11	0.17	3.11E-11
1.00	5.38E-11	1.00	5.38E-11	2.60	3.41E-11	0.33	3.49E-11
1.63	5.36E-11	1.63	4.95E-11	2.62	3.41E-11	0.73	2.84E-11
1.85	5.33E-11	1.85	5.67E-11	3.20	4.17E-11	1.53	3.58E-11
1.95	5.31E-11	1.95	5.14E-11	4.04	4.17E-11	1.74	3.44E-11
2.01	5.17E-11	2.01	5.36E-11	5.07	4.17E-11	2.51	3.53E-11
2.47	5.45E-11	2.47	5.31E-11	5.09	3.75E-11	4.26	3.46E-11
3.10	5.64E-11	3.10	5.61E-11	7.79	3.75E-11	4.80	3.76E-11
3.19	5.78E-11	3.19	5.75E-11	7.81	3.75E-11	6.35	3.87E-11
3.59	5.63E-11	3.59	5.70E-11	8.63	7.23E-11	7.88	4.11E-11
3.66	5.84E-11	3.66	5.76E-11	10.16	7.23E-11	9.42	3.52E-11
3.74	5.61E-11	3.74	5.72E-11	11.67	2.36E-11	10.95	3.69E-11
3.81	5.62E-11	3.81	5.63E-11	11.69	7.23E-11	12.48	3.29E-11
4.50	6.22E-11	4.50	5.77E-11	11.69	2.36E-11	14.01	3.28E-11
5.05	5.69E-11	5.05	5.79E-11	13.43	7.42E-11	15.54	3.25E-11
5.25	5.73E-11	5.25	5.69E-11	13.45	7.42E-11	17.07	3.41E-11
5.61	6.22E-11	5.61	6.08E-11	13.92	2.36E-11	18.60	3.58E-11
5.91	5.67E-11	5.91	5.97E-11	14.76	2.36E-11	20.14	3.10E-11
6.00	5.56E-11	6.00	5.90E-11	15.23	7.49E-11	21.73	3.28E-11
6.24	5.51E-11	6.24	5.56E-11	15.25	7.49E-11	23.21	3.55E-11
6.49	5.55E-11	6.49	5.46E-11	16.27	7.49E-11	24.75	3.89E-11
6.55	5.17E-11	6.55	5.82E-11	17.05	4.21E-11		
6.82	5.32E-11	6.82	5.22E-11	17.07	4.21E-11		
6.90	5.21E-11	6.90	5.14E-11	17.83	4.21E-11		
6.96	4.96E-11	6.96	5.48E-11	18.86	6.32E-11		
7.80	5.57E-11	7.80	5.24E-11	18.88	6.32E-11		
7.89	5.39E-11	7.89	5.13E-11	19.37	9.37E-11		
7.95	5.69E-11	7.95	5.07E-11	20.90	3.24E-11		
8.05	5.72E-11	8.05	5.57E-11	22.43	8.28E-11		
8.56	5.01E-11	8.56	4.77E-11	23.86	8.62E-11		

9.16	5.28E-11	9.16	4.80E-11	25.40	7.11E-11				
9.25	4.88E-11	9.25	5.43E-11	25.54	8.62E-11				
9.31	5.56E-11	9.31	5.49E-11	26.21	4.77E-11				
9.36	5.08E-11	9.36	5.28E-11						
9.44	5.32E-11	9.44	4.88E-11						
9.80	5.69E-11	9.80	5.32E-11						
9.86	5.48E-11	9.86	5.00E-11						
9.95	5.31E-11	9.95	5.67E-11						
10.04	6.21E-11	10.04	5.69E-11						
10.10	6.13E-11	10.10	5.48E-11						
10.19	6.04E-11	10.19	5.31E-11						
10.25	6.41E-11	10.25	6.21E-11						
10.34	8.23E-11	10.34	6.13E-11						
10.40	6.97E-11	10.40	6.04E-11						
10.74	1.16E-10	10.74	7.39E-11						
10.89	7.24E-11	10.89	6.78E-11						
10.94	7.12E-11	10.94	7.43E-11						
11.00	6.42E-11	11.00	1.16E-10						
11.07	7.74E-11	11.07	7.24E-11						
11.46	7.94E-11	11.46	7.15E-11						
11.54	7.32E-11	11.54	5.75E-11						
11.61	7.25E-11	11.61	7.95E-11						
11.85	7.86E-11	11.85	7.94E-11						
11.91	8.52E-11	11.91	7.32E-11						
12.01	7.16E-11	12.01	7.25E-11						
12.09	6.90E-11	12.09	7.86E-11						
12.15	6.02E-11	12.15	8.52E-11						
12.22	6.11E-11	12.22	7.16E-11						
12.27	6.29E-11	12.27	6.90E-11						
12.60	4.50E-11	12.60	5.15E-11						
12.66	4.66E-11	12.66	4.82E-11						
13.06	4.91E-11	13.06	4.26E-11						
13.14	4.61E-11	13.14	4.50E-11						
13.30	3.89E-11	13.30	4.66E-11						
13.36	3.13E-11	13.36	4.91E-11						
13.45	3.11E-11	13.45	4.61E-11						
13.86	2.44E-11	13.86	2.77E-11						
13.94	2.71E-11	13.94	2.64E-11						
14.26	3.66E-11	14.26	2.42E-11						
14.49	3.44E-11	14.49	2.44E-11						

14.56	3.48E-11	14.56	2.71E-11				
14.65	2.94E-11	14.65	3.66E-11				
14.71	3.19E-11	14.71	3.44E-11				
15.19	3.89E-11	15.19	3.78E-11				
15.25	3.93E-11	15.25	3.87E-11				
15.34	3.69E-11	15.34	3.84E-11				
15.40	3.64E-11	15.40	3.89E-11				
15.45	3.67E-11	15.45	3.93E-11				
15.50	3.52E-11	15.50	3.69E-11				
15.56	3.75E-11	15.56	3.64E-11				
15.86	4.11E-11	15.86	3.67E-11				
15.95	3.85E-11	15.95	3.52E-11				
16.01	4.02E-11	16.01	3.75E-11				
16.61	2.75E-11	16.61	3.48E-11				
16.70	2.52E-11	16.70	2.92E-11				
16.79	2.34E-11	16.79	2.79E-11				
16.85	2.44E-11	16.85	2.75E-11				
16.94	2.44E-11	16.94	2.52E-11				
17.00	2.43E-11	17.00	2.34E-11				
17.55	2.91E-11	17.55	2.66E-11				
17.76	2.96E-11	17.76	2.69E-11				
17.84	3.16E-11	17.84	2.74E-11				
17.90	3.05E-11	17.90	2.91E-11				
17.99	3.13E-11	17.99	2.96E-11				
18.20	3.01E-11	18.20	3.16E-11				
18.29	3.08E-11	18.29	3.05E-11				
18.34	2.78E-11	18.34	3.13E-11				
19.21	2.12E-11	19.21	2.20E-11				
19.26	2.50E-11	19.26	2.15E-11				
19.30	2.42E-11	19.30	1.86E-11				
19.54	2.56E-11	19.54	2.12E-11				
20.00	1.92E-11	20.00	2.57E-11				
21.46	2.97E-11	21.46	2.55E-11				
21.55	3.17E-11	21.55	2.75E-11				
21.61	2.99E-11	21.61	2.84E-11				
21.84	3.74E-11	21.84	2.97E-11				
21.91	4.09E-11	21.91	3.17E-11				
21.96	4.44E-11	21.96	2.99E-11				
22.21	4.80E-11	22.21	3.74E-11				
22.29	4.43E-11	22.29	4.09E-11				

23.15	3.99E-11	23.15	4.36E-11
23.25	4.67E-11	23.25	4.75E-11
23.35	4.82E-11	23.35	5.19E-11
23.45	5.25E-11	23.45	3.99E-11
23.50	4.97E-11	23.50	4.67E-11
23.61	4.47E-11	23.61	4.82E-11
23.70	4.31E-11	23.70	5.25E-11
23.75	4.36E-11	23.75	4.97E-11
23.85	5.41E-11	23.85	4.47E-11
23.95	5.00E-11	23.95	4.31E-11
24.30	4.97E-11	24.30	4.36E-11

Supp. Table 5. Calculated radiolytic H_2 production rates at North Pacific and North Atlantic sites. mbsf = meters beneath the seafloor.

**Lithology**	**G(H_2)-γ** Molecules H_2 MeV^{-1}	**G(H_2)-α** Molecules H_2 MeV^{-1}	**U** (ppm)	**Th** (ppm)	**K** (ppm)	**Density** (g/cm^3)
Abyssal clay	8455	248464	2.51	11.21	18748	2.70
Siliceous ooze	18250	200750	1.82	10.44	24790	2.30
Calcareous marl	6200	157300	1.16	9.04	1100	2.44
Calcareous ooze	4800	62000	0.77	0.53	684	2.70
Lithogenous	2800	146100	2.70	12.00	20753	2.70
Other	2800	146100	2.70	12.00	20753	2.70

Supp. Table 6. Lithology-specific sets of parameters used to calculate global marine sedimentary radiolytic H_2 production.

Site	Depth (mbsf)	DIC production rate (mol DIC/yr/m^3)	First standard deviation (mol DIC/yr/m^3)
1225	0-168.3	2.33E-07	1.96E-07
	168.9-291.6	6.74E-07	3.24E-07
1226	0-8.96	1.35E-04	8.44E-06
	9.13-35.6	1.33E-05	6.37E-06
	35.8-121.2	3.30E-06	1.60E-06
	121.4-271.8	6.09E-07	4.41E-06
	271.9-304.8	1.13E-05	7.60E-06
	356-417.7	1.50E-06	1.91E-06
1230	0-15.6	2.46E-03	1.18E-03
	15.8-44.2	3.48E-04	9.30E-04
	44.3-161.6	8.21E-05	1.10E-04
U1343	0-2.39	1.12E-02	1.15E-02
	5.43-8.5	7.06E-03	5.56E-03
	8.7-14.5	6.99E-04	3.63E-03
	14.8-29.1	2.08E-04	5.21E-04
	29.3-521	7.10E-05	6.79E-05
U1345	0-8.3	2.93E-03	3.61E-04
	8.6-12.8	4.97E-04	1.01E-03
	13.1-25.4	2.20E-04	1.82E-04
	25.7-139.4	7.76E-06	1.15E-04
U1371	0-3.0	4.84E-04	4.60E-04
	11.6-24.8	6.72E-06	2.57E-05
	25.2-41.0	2.35E-07	1.21E-05
	63.7-85.5	6.36E-06	6.01E-06
	108.1-130.1	3.70E-06	7.10E-06

Supp. Table 7. Vertical distribution of dissolved inorganic carbon (DIC) production rates (net organic-fueled respiration) at North Pacific, Equatorial Pacific and South Pacific and Bering Sea sites. mbsf = meters beneath the seafloor.

	Ocean Basin	Site	Basement Age (Ma)	Sediment thickness (m)	Sediment accumulation rate (m/Ma)
Oxic sites	South Pacific	IODP Site U1365	100	75	0.75
		IODP Site U1366	95	30	0.32
		IODP Site U1367	33.5	27	0.81
		IODP Site U1368	13.5	16	0.81
		IODP Site U1369	58	16	0.28
		IODP Site U1370	75	68	0.91
		IODP Site U1371	75	130	1.73
	North Atlantic	KN223 Site 11	70	100	1.43
		KN223 Site 12	90	98	1.09
	North Pacific	KN195-EQP10	68.5	100	1.46
		KN195-EQP11	88.7	100	1.13
Anoxic sites	South Pacific	U1371	75	130	1.73
	Equatorial Pacific	ODP Site 1225	11.3	318	18
		ODP Site 1226	17.1	439	24
	Peru Trench	ODP Site 1230	−	626.5	20
	Peru Basin	ODP Site 1231	37.3	91.5	15
	Bering Sea	IODP Site U1343	−	6960	235
		IODP Site U1345	−	1450	280

Supp. Table 8. Basement age, sediment thickness and mean sediment accumulation rate at the North Pacific, South Pacific, and North Atlantic coring sites included in this study.
